# Analysis of Factors Influencing the Bonding Performance at the FFRP-Timber Interface

**DOI:** 10.3390/ma19050991

**Published:** 2026-03-04

**Authors:** Yuanyuan Xia, Weilong Zhang, Jianbo Tian, Yangyang Xia

**Affiliations:** 1 School of Civil Engineering and Architecture, Xi’an University of Technology, Xi’an 710048, China; xiayuanyuan@xaut.edu.cn (Y.X.); 2240721212@stu.xaut.edu.cn (W.Z.); tianjianbo@xaut.edu.cn (J.T.); 2 School of Engineering, Tibet University, Lhasa 850000, China

**Keywords:** flax fiber reinforced polymer, interfacial stress and strain distribution, ultimate bearing capacity, bond-slip relationship

## Abstract

Flax Fiber Reinforced Polymer (FFRP), as a green material with nonlinear large deformation characteristics, is used in the reinforcement of timber structures. Due to the similar elastic moduli of FFRP, adhesive, and timber, stress concentration at the interface is significantly reduced, demonstrating favorable interfacial performance. This study investigates the effects of adhesive layer thickness and FFRP laminate thickness on the strain distribution, bond-slip relationship, and stress distribution at the FFRP-timber interface through two different types of single-lap shear tests, thereby revealing the bonding mechanism at the FFRP-timber interface. The results show that both the ultimate load and the ultimate strain at the loaded end decrease with increasing adhesive thickness. For instance, increasing the adhesive thickness from 0.5 mm to 3 mm led to a 68.6% reduction in peak interfacial shear stress. The thickness of the adhesive has a minor influence on the overall trend of the bond-slip relationship curve for the FFRP-timber interface, with the curve consisting of an ascending branch, a descending branch, and a horizontal plateau. The distribution patterns of interfacial shear stress for different adhesive layer thicknesses are similar: at the initial loading stage, the maximum shear stress appears at the loaded end and gradually decreases toward the free end; as the load increases, the peak shear stress shifts from the loaded end toward the free end. With an increase in the number of fiber layers in the FFRP laminate, the strain transfer efficiency first increases and then decreases, reaching its maximum when the number of fiber layers reaches 30. The maximum stress increases with the number of FFRP fiber layers, and the stress transfer efficiency peaks at 30 layers.

## 1. Introduction

Timber structures are treasures of Chinese culture and historical heritage, possessing numerous structural advantages. These include the separation of enclosure structures from load-bearing systems, high seismic resistance, the use of green raw materials that help reduce carbon dioxide emissions [1,2], ease of sourcing and construction, and low cost [3]. These benefits have driven a significant rise in new timber construction. With technological advancements, issues such as the low load-bearing capacity and poor corrosion resistance of timber have been largely addressed. Processed materials like glulam and composite timber structures can achieve load-bearing capacities comparable to steel. However, during service, timber structures are susceptible to factors such as insect damage, fungal decay, and natural disasters [4]. Over time, components inevitably experience aging, loosened joints, and deformation, which degrade the overall load-bearing capacity, making the reinforcement of timber structures an urgent issue.

Externally bonded Fiber Reinforced Polymer (FRP) is an effective method for repairing and strengthening damaged historical timber structures. In recent years, Carbon Fiber Reinforced Polymer (CFRP) has been successfully applied in the restoration and reinforcement of ancient structures [4,5,6,7,8,9,10,11,12,13]. However, the tensile strength and modulus of CFRP are far superior to those of timber, and its low elongation at break leads to incompatibility in load-deformation behavior with the reinforced timber, often causing premature interfacial failure. In comparison, FFRP exhibits mechanical properties closer to timber, enabling more effective interfacial stress transfer. Although the mechanical properties of FFRP are lower than those of CFRP, it demonstrates higher efficiency in reinforcing timber beams [14]. The fundamental mechanisms governing FRP-timber interfacial behavior have been systematically reviewed by Wang et al. [15], who synthesized knowledge on stress transfer mechanisms, shear stress distribution patterns, and failure modes across multiple testing methodologies. Similarly to other externally bonded FRP techniques, the performance of the FFRP-timber interface is crucial to the effectiveness of the FFRP reinforcement.

Currently, numerous studies have investigated the effect of adhesive bondline thickness on the interfacial performance of FRP-concrete and FRP-steel interfaces. However, research on interfacial bond performance with timber substrates remains scarce. Some scholars suggest that the influence of adhesive thickness on the FRP-timber interface may be referenced from studies on FRP-concrete interfaces. Dai et al. [16] conducted experiments on FRP-strengthened concrete structures. The results indicated that reducing the shear stiffness of the adhesive could enhance the bond performance of the FRP-concrete interface. Adhesives with lower stiffness, offering better toughness, can increase the effective bond length. Tamura et al. [17] performed beam tests and concluded that as the adhesive bondline thickness increases, the bond strength of the FRP-concrete interface gradually improves, albeit with a small margin, and the effective bond length also increases. Mazumdar et al. [18] studied the bond behavior of composite bonded joints under load. They found that the failure load of the joint increases with greater lap length; when the adhesive thickness is less than 0.33 mm, the joint failure load is positively correlated with the adhesive layer thickness, but becomes negatively correlated when the thickness exceeds 0.33 mm. Peng et al. [19] conducted single-lap shear tests on CFRP-concrete, with concrete grade, bond length, and adhesive thickness as variables. The results showed that both adhesive thickness and concrete grade affect interfacial bond performance. As the adhesive thickness (or concrete grade) increases, the ultimate interfacial load-bearing capacity also improves. The interfacial fracture energy increases with greater adhesive bondline thickness. Guo et al. [20] performed CFRP-concrete single-lap shear tests. The results indicated that for lower concrete grades, increasing the adhesive bondline thickness leads to a higher ultimate load-bearing capacity, reaching a maximum at 3 mm adhesive thickness. For higher concrete grades, when the adhesive thickness is less than 3 mm, the ultimate load-bearing capacity is positively correlated with adhesive thickness; when the thickness exceeds 3 mm, the rate of increase in ultimate capacity diminishes with further thickness increase.

Regarding FFRP reinforcement of timber, much research has focused on the number of FFRP fiber layers. As the number of flax fiber layers increases, the flexural load-bearing capacity of timber beams also improves, with FFRP demonstrating reinforcement effectiveness comparable to other FRPs [21]. Compared to three-layer FFRP, five-layer FFRP can enhance the reinforcement effect on timber by 27% [22]. Monica. V investigated the bending capacity and stiffness of cross-laminated timber (CLT) reinforced with single and double layers of flax fiber. The results showed significant improvements in both load-bearing capacity and stiffness for the reinforced three-layer CLT [23]. Borri. A studied the load-bearing capacity and stiffness of timber beams reinforced with 1–7 layers of natural fibers. The findings indicated that flax fiber reinforcement outperforms basalt fiber reinforcement [24]. The aforementioned studies demonstrate that research using low-layer-count FFRP for timber reinforcement has been conducted. However, there appears to be a lack of research on the fabrication techniques for high-layer-count FFRP in timber reinforcement.

While previous studies have primarily focused on low-ply FFRP (1–7 layers) for timber reinforcement, this study extends the investigation to high-ply configurations (up to 50 layers), which are critical for understanding the interfacial behaviour under realistic structural applications. Furthermore, this paper provides a quantitative comparison of adhesive thickness effects and proposes a classification of interfacial stress distribution patterns, offering a more nuanced understanding of the bond mechanism than previously available.

This study tests the following hypotheses: (1) Increasing adhesive thickness beyond 1.5 mm leads to a reduction in interfacial load capacity due to increased adhesive stiffness and reduced deformation energy absorption; (2) There exists an optimal number of FFRP plies (around 30) that maximizes interfacial strain transfer efficiency and ductility.

## 2. Materials and Methods

### 2.1. Materials

#### 2.1.1. Timber

The timber species used was Russian pine, supplied by Shaanxi Xinyaosen Wood Industry Co., Ltd., Xi’an, China. The basic material parameters of the timber are listed in Table 1. The Russian pine sawn timber exhibits straight surface grain, contains live knots, and is free of defects, corresponding to first-grade quality. The length direction of the specimens is aligned with the grain direction.

#### 2.1.2. FFRP

**Figure 1:**
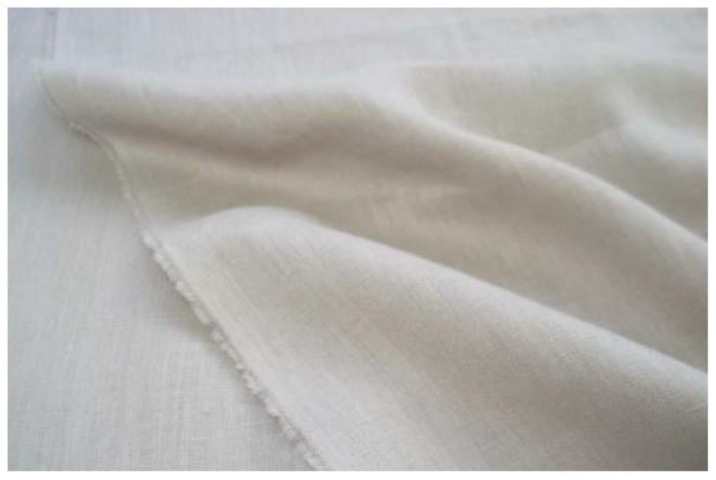
Flax fiber textile.

The FFRP plates used in this test were fabricated by bonding flax fiber fabric with an epoxy impregnating adhesive, designated as TS. The flax fiber fabric was supplied by Harbin Changli Linen Factory, as shown in Figure 1. The mechanical properties of the flax fiber composite plates were tested according to the Chinese standard GB/T3354-2014 [25]. The obtained physical and mechanical properties are presented in Table 2.

#### 2.1.3. Adhesive

The adhesive employed was the epoxy structural adhesive T1, produced by Shandong Dagong Composite Materials Co., Ltd., Linyi, China. The selection of T1 epoxy adhesive was based on its suitability for timber bonding applications. Miao et al. [26] emphasized that adhesive selection critically influences FRP-timber joint performance. This adhesive is a two-component system consisting of Part A (epoxy resin) and Part B (hardener). The specific parameters are listed in Table 3.

### 2.2. Experiment on the Influence of Adhesive Thickness on Interfacial Bonding Performance

#### 2.2.1. Experimental Design

**Figure 2:**
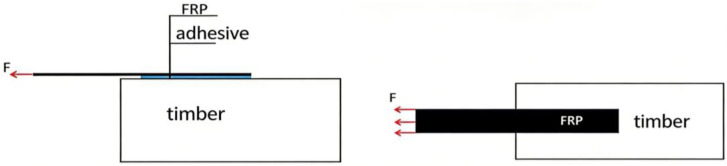
FFRP-timber structure specimen model.

**Figure 3:**
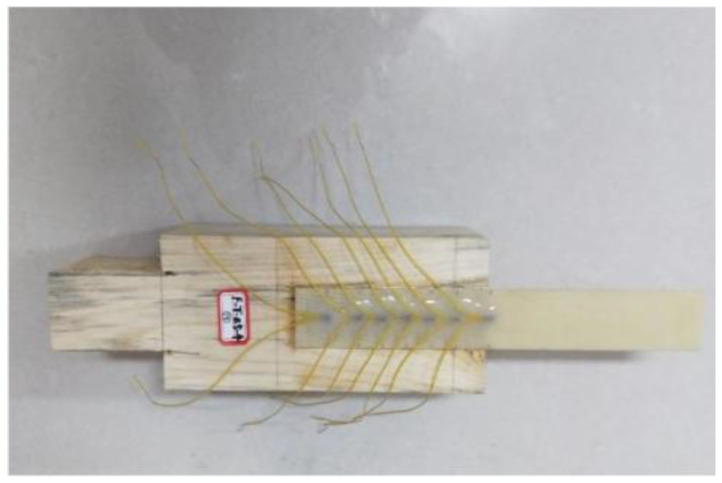
Attach a strain gauge to the test piece.

To investigate the influence of adhesive bondline thickness on the interfacial bonding performance between FFRP plates and timber structures, single-lap shear tests were conducted using existing laboratory equipment. Wooden specimens measuring 80 mm × 80 mm × 200 mm were prepared. An FFRP plate was bonded to one side of the specimen, which had been surface-treated by sanding. An unbonded region of 20 mm was reserved at the loading end. The specimen model is shown in Figure 2. A crucial aspect of the experimental design is the bond length. An excessively long bond length leads to material waste, while an insufficient bond length fails to ensure effective bonding, resulting in inaccurate measurements of interfacial bond strength. Previous tests have indicated that the effective bond length for the FFRP-timber interface falls within the range of 95 mm to 105 mm [14]. Therefore, a bond length of 100 mm was selected for this study. Another key parameter in the design is the adhesive thickness. Xia [27] experimentally concluded that an adhesive layer thickness exceeding 3 mm can affect the failure mode of the interface. Conversely, a thickness below 0.5 mm is prone to significant relative errors during specimen fabrication. Consequently, adhesive layer thicknesses of 0.5 mm, 1 mm, 2 mm, and 3 mm were chosen for this investigation. The relevant parameters of the test specimens are listed in Table 4. Prepared specimens are shown in Figure 3.

#### 2.2.2. Test Method

**Figure 4:**
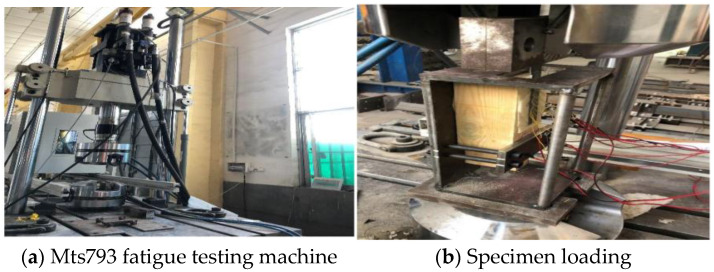
Loading mode of FFRP-timber structure specimen.

**Figure 5:**
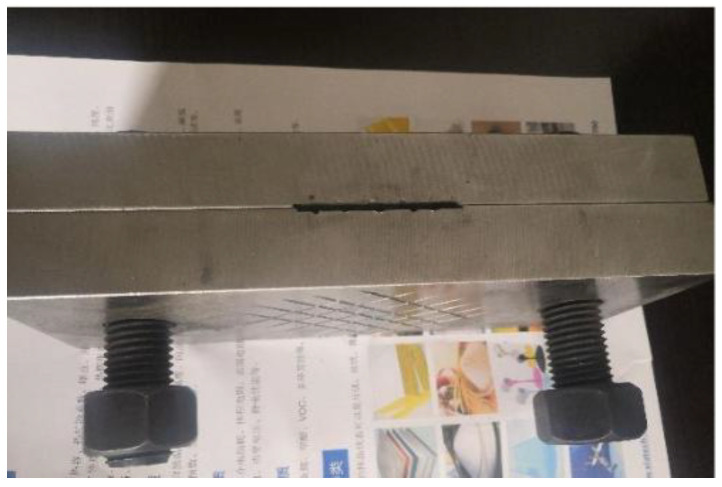
Steel structure fixture.

This study employed the single-lap shear test to evaluate the bonding performance of the FFRP-timber interface. The strain values on the FFRP plate were recorded simultaneously during loading. The loading configuration for the specimen is shown in Figure 4. An MTS793 electro-hydraulic servo fatigue testing machine (MTS Systems Corporation, Eden Prairie, MN, USA) served as the loading apparatus. To prevent eccentricity issues during loading, caused by the relatively wide grips of the testing machine and the thin FFRP plates, a steel structural fixture was utilized, as illustrated in Figure 5. A pre-loading sequence was performed before formal testing to avoid undesirable results due to a sudden application of external force. The tests were conducted at a constant loading rate of 1 mm/min.

During the single-lap shear tests, the applied force was automatically recorded by the fatigue testing machine, and the strain values were obtained by connecting the bonded strain gauges to a data acquisition system. The use of strain gauges to monitor interfacial behavior follows established practices for characterizing bonded joints. Almonti et al. [28] recently demonstrated similar single-lap shear testing methodologies for evaluating interfacial performance in composite coatings, where detailed strain distribution analysis provided insights into stress transfer mechanisms. Their approach to correlating material properties with interfacial behavior informs the analytical framework adopted in the present study for FFRP-timber interfaces.

### 2.3. Experiment on the Influence of FFRP Plate Thickness on Interfacial Bonding Performance

#### 2.3.1. Experimental Design

**Figure 6:**
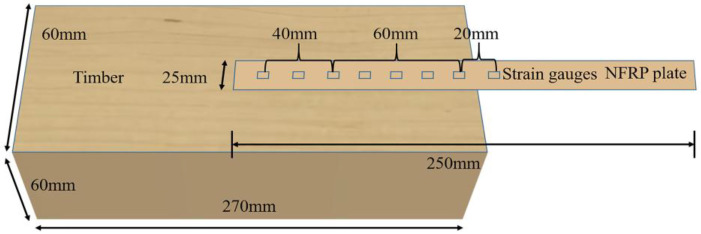
Single-lap shear specimen design.

**Figure 7:**
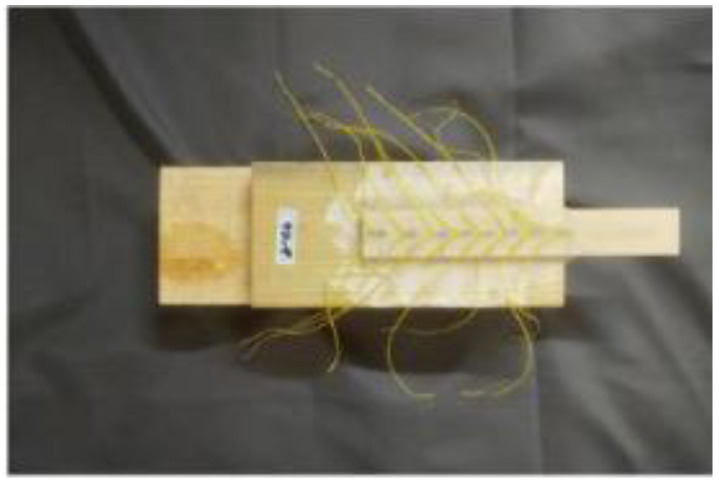
Attach a strain gauge to the test piece.

To investigate the influence of FFRP plate thickness on the interfacial bonding performance between the FFRP plate and the timber structure, FFRP plates with 10, 20, 30, 40, and 50 layers were fabricated using the autoclave molding process. The designed single-lap shear specimen is illustrated in Figure 6. The timber substrate dimensions were b × h × l = 60 mm × 60 mm × 270 mm. The epoxy structural adhesive T1 was used for bonding. Strain gauges were attached starting from a point 20 mm before the bonded region (i.e., on the unbonded part of the FFRP at the loading end) and along the FFRP-timber interface. Specific gauge locations were at distances of 15 mm, 30 mm, 45 mm, 60 mm, 80 mm, and 100 mm from the start of the bonded section. The calculation method for the elastic modulus of the FFRP plates refers to ASTM D3039 [29], where the slope of the linear region within the strain range of 0–0.001 is taken as the elastic modulus of the material. It was observed that the modulus of FFRP varies significantly with the number of fiber layers. The tensile test results for all FFRP specimens are presented in Table 5. Prepared specimens are shown in Figure 7.

A comparison of FFRP plates with different numbers of fiber layers revealed that, as the number of fiber layers increased, both the tensile strength and modulus decreased, and the elongation at break generally exhibited a decreasing trend. The increase in the number of fiber layers led to a reduction in elongation at break and a decrease in the strength of the plate. The potential reasons include: increased fiber waviness due to the higher layer count; an increase in resin-rich areas, which contributes to strength reduction; and the accumulation of voids or defects during the pressing process.

#### 2.3.2. Test Method

**Figure 8:**
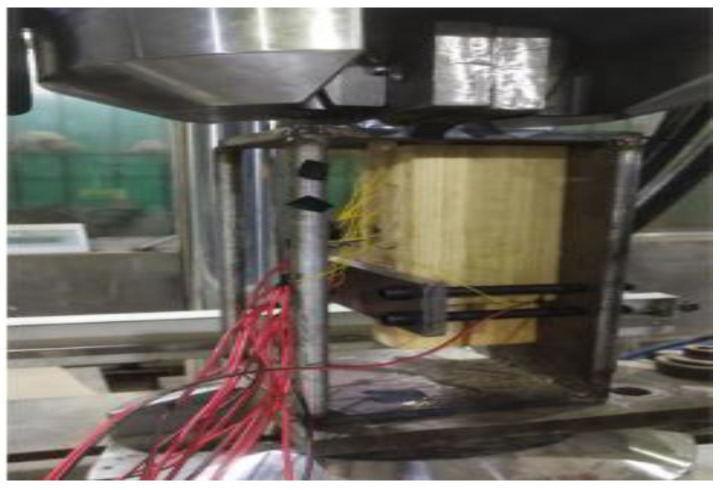
Specimen Loading.

During the single-lap shear tests, an MTS793 electro-hydraulic servo fatigue testing machine was employed as the loading apparatus. To ensure that the specimens were not subjected to excessive initial load, which could lead to unstable test data, a small preload was applied prior to formal loading. The specimens were loaded under displacement control at a constant rate of 1 mm/min. Other parameters, such as loading frequency, safety limits, and data storage paths, were configured accordingly. Before testing, the displacement and load readings of the testing machine were zeroed. The specimen was then mounted on the single-lap shear test fixture, and the strain gauges were connected to the data acquisition system. During loading, the deformation of the specimen was monitored until failure occurred, at which point the loading program was manually stopped, followed by termination of data acquisition. The loading configuration is shown in Figure 8.

Throughout the test, the applied load was automatically recorded by the testing machine’s control system. Strain data were acquired automatically via a dynamic data acquisition unit connected in a quarter-bridge configuration to the strain gauges bonded on the FFRP surface, at a sampling frequency of 2 Hz. The acquisition processes were synchronized. To ensure a one-to-one correspondence between load and strain readings, strain data acquisition was initiated before starting the loading sequence of the testing machine.

## 3. Effect of Adhesive Thickness on the Interfacial Bonding Performance

### 3.1. Ultimate Load-Bearing Capacity and Failure Mode of the Interface

In the tests, T1 adhesive was used with bond line thicknesses of 0.5 mm, 1 mm, 2 mm, and 3 mm, respectively, to conduct single-lap shear tests. The test results are presented in Table 6.

**Figure 9:**
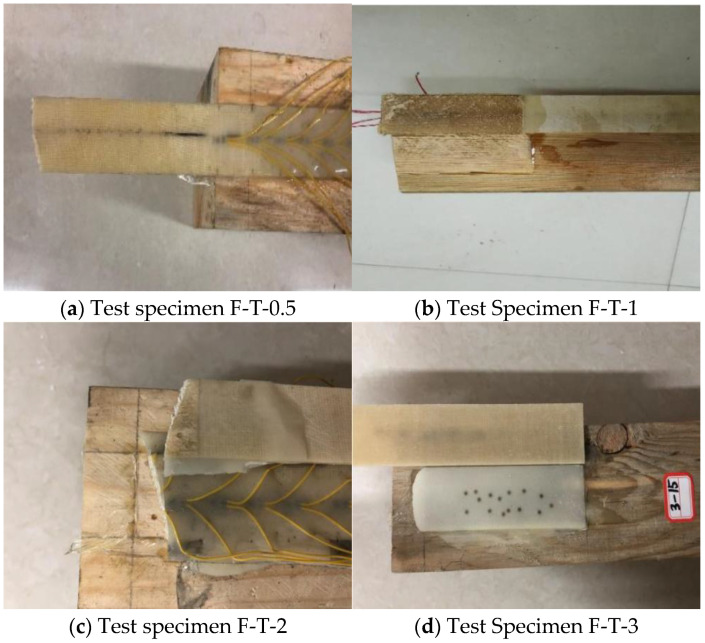
Interface failure mode.

The experimental results indicate that under identical conditions, different specimens exhibited relatively similar ultimate load-bearing capacities at failure. Therefore, only the average values of the ultimate load-bearing capacity are presented in Table 6. A comparison of the results for specimens using T1 adhesive with varying adhesive layer thicknesses revealed that as the adhesive layer thickness increased, the ultimate load-bearing capacity of the interface decreased, with the extent of reduction varying. Specifically, when the adhesive layer thickness increased from 0.5 mm to 1 mm, the ultimate load-bearing capacity decreased by approximately 6.1%. When the thickness increased from 1 mm to 2 mm, the capacity decreased by about 12.3%. Furthermore, when the thickness reached 3 mm, the ultimate load-bearing capacity was 31.8% lower than that at 2 mm. These findings indicate that the thickness of the adhesive layer has a significant influence on the ultimate load-bearing capacity of the T1 adhesive interface. Two failure modes were observed during the experiments: fracture of the FFRP plate and peeling failure of the wood, as illustrated in Figure 9. Specimen F-T-0.5 exhibited failure due to FFRP plate fracture, as shown in Figure 9a. This suggests that at an adhesive layer thickness of 0.5 mm, the resin demonstrated excellent permeability, resulting in high interfacial bond strength and ultimately leading to fracture of the FFRP plate. For specimen F-T-1, the failure mode was characterized by peeling failure between the FFRP plate and the wood, as depicted in Figure 9b. The wood surface exhibited grooves approximately 1 mm in depth, and the width of the wood on the fiberboard matched that of the grooves, indicating that the specimen fabrication process was appropriate. The high permeability of the resin contributed to a strong bond at the interface, allowing the full performance of both the FFRP plate and the adhesive. Specimen F-T-2 displayed a mixed-mode failure, as shown in Figure 9c. In the case of specimen F-T-3, failure occurred via debonding at the interface between the FFRP plate and the upper surface of the adhesive layer, as illustrated in Figure 9d. Upon failure, no wood debris adhered to the FFRP plate, which was attributed to inadequate surface preparation during specimen fabrication, leading to premature interfacial failure. The failure mode observed in specimen F-T-1 represents the ideal experimental outcome.

### 3.2. Interfacial Strain Distribution

**Figure 10:**
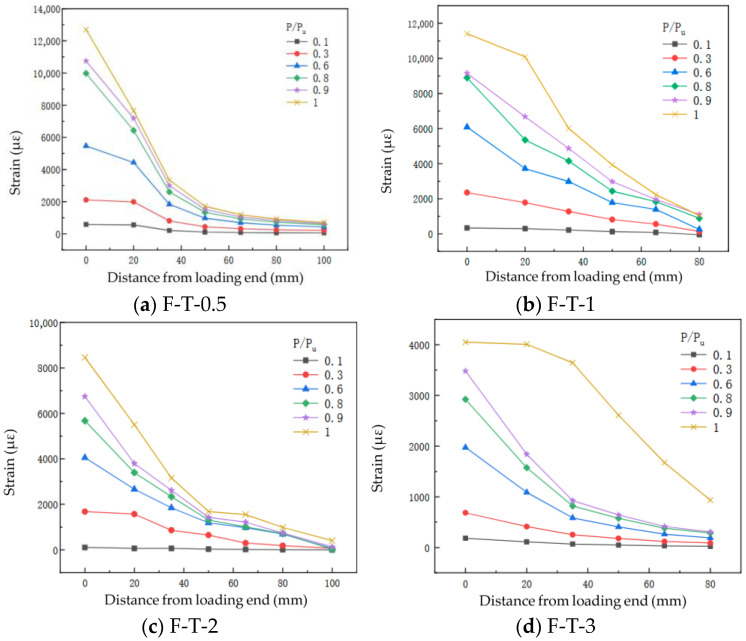
FFRP sheet strain distribution.

Figure 10 shows the axial strain distribution in the flax fiber composite plates for the four adhesive layer thicknesses. The strain in the plates was recorded by the data acquisition system. The magnitude of the plate strain can be used to analyze the stress state at the interface.

For specimen F-T-0.5, the axial strain distribution of the FFRP plate during loading is shown in Figure 10a, with the loading end defined as the origin, mathematically expressed as x = 0 mm. From Figure 10a, it can be observed that when the load on the FFRP plate is relatively small (P = 0.1 Pu), the strain gradually decreases from the loading end toward the free end. When the strain propagates to x = 100 mm, the load is primarily carried by the bonded region within approximately 35 mm from the loading end. Specific strain values of the FFRP plate are listed in Table 7. As the load increases (P = 0.3 Pu), the axial strain of the FFRP plate also increases. At this stage, the load is mainly borne by the bonded region within about 50 mm, and the axial strain of the FFRP plate exhibits a gradually decreasing trend from left to right. When the load reaches P = 0.6 Pu, the bonded region bearing the load expands to approximately 65 mm. At a load equal to 0.8 Pu, the load-bearing bonded region further extends to about 80 mm. Throughout the entire loading process of specimen F-T-0.5, no softening or debonding phenomena were observed in the bonded area near the loading end. In this study, the onset of softening was identified by a decrease in the strain gradient between adjacent gauges near the loaded end, indicating local damage accumulation without complete loss of load transfer capability. Debonding was defined when the strain difference between two adjacent points became negligible, suggesting that the interface in that region could no longer sustain shear stress. When the load equals 1.0 Pu, the strain at x = 100 mm is 703.26 microstrain (με). Given that the free end is located at x = 120 mm, it can be concluded that the entire designed bonded region in this test was subjected to load.

Figure 10b shows the axial strain distribution of the FFRP plate for specimen F-T-1. When the load level P/Pu ≤ 0.8, the strain distribution of the FFRP plate is essentially consistent with that of specimen F-T-0.5. At a load equal to 0.9 Pu, the strain value at x = 0 mm is 9151.94 με, and the strain at x = 20 mm is 6676.04 με, resulting in a difference of 2475.90 με. In comparison, the difference at a load of 0.8 Pu was 3545.68 με. The decrease in this difference indicates that the bond within this region (x = 0 mm to 20 mm) has entered a softening stage. When the load reaches the ultimate load (i.e., P/Pu = 1), as can be seen from Table 8, the difference becomes 1314.07 με. This further reduction (from 2475.90 με to 1314.07 με) suggests that softening occurred around the interface in this area, although debonding failure did not occur. At the ultimate load level (1 Pu), the strains at x = 80 mm for specimens F-T-1 and F-T-0.5 are 1044.26 με and 918.24 με, respectively. This indicates that the external force in specimen F-T-1 is transferred over a larger area along the bonded region, suggesting that the effective bond length of the interface can increase with greater adhesive layer thickness.

The axial strain distribution of the flax fiber-reinforced polymer (FFRP) plate for specimen F-T-2 is shown in Figure 10c. The strain curves obtained during loading also exhibit a gradually decreasing trend from left to right. Moreover, no softening or debonding of the adhesive around the loaded end was observed throughout the loading process of specimen F-T-2. The strain values of the FFRP plate under different load levels are given in Table 9. At a load level of 0.9 Pu, the strain difference between x = 0 mm and x = 20 mm is 2953.40 με. When the load reaches 1.0 Pu, the strain difference between the loaded end at 0 mm and 20 mm is 2960.77 με, which is close to that at 0.9 Pu with no noticeable decreasing trend. Additionally, the strain at the free end (x = 100 mm) is 403.96 με. Given the relatively small strain value, it can be inferred that the load is transmitted up to x = 120 mm, indicating that the bonded region within 100 mm is fully engaged in load transfer.

The axial strain distribution of the flax fiber-reinforced polymer (FFRP) plate for specimen F-T-3 is shown in Figure 10d. When P/Pu is less than 0.9, the axial strain distribution of the FFRP plate also exhibits a gradual decrease from left to right. At P/Pu = 1, the strain at x = 0 mm is 4051.98 με, while the strain at x = 20 mm is 4011.12 με. As presented in Table 10, the difference between these two values is minimal. This indicates that softening occurred near the loaded end, followed by interface debonding in that region. Consequently, the load was primarily transferred and borne within the range of x = 20 mm to 100 mm.

Analyzing the strain distribution patterns in Figure 10, the main types of strain distribution curves observed are as follows:

(1) Strain distribution curves obtained from specimens that did not experience debonding from loading until failure, as shown in Figure 10a,c. These can be regarded as incompletely developed strain distribution curves.
(2) As the load continuously increased, the strain distribution curves under higher load levels became relatively flatter compared to those under lower load levels. The full and developed shape of these strain distribution curves reflects the occurrence of debonding, as illustrated by the strain curves at P/Pu = 1 in Figure 10b,d.

**Figure 11:**
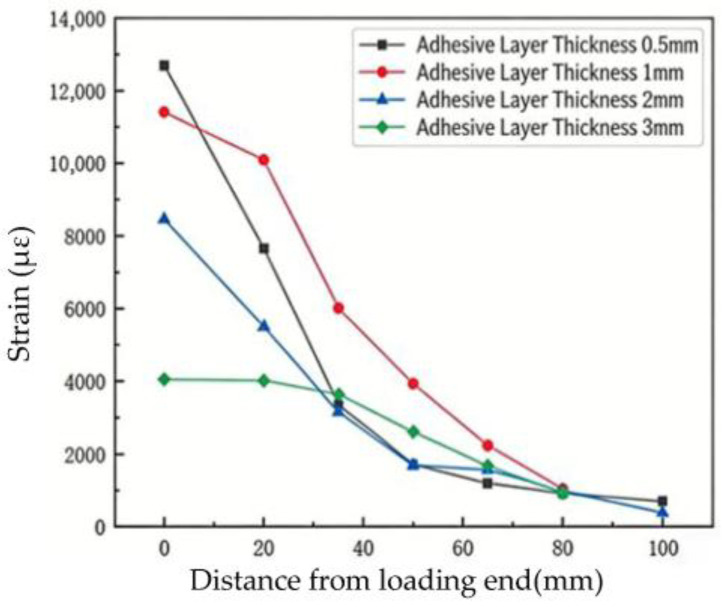
Strain value under ultimate bearing capacity of each specimen.

Figure 11 shows the strain distribution diagrams of the FFRP-to-timber interface specimens at ultimate load. It can be observed that for specimens with adhesive thicknesses of 0.5 mm, 1 mm, and 2 mm, the strain curve exhibits a steep slope in the initial stage. This indicates that when the ultimate load is reached, the entire bonded region participates in load transfer. In contrast, the strain curve for the specimen with a 3 mm adhesive thickness shows a near-zero slope at the beginning, suggesting that interface separation has occurred around the loaded end, and the interface can no longer sustain shear stress. From this, it can be concluded that the ultimate load capacity of the interface decreases with increasing adhesive thickness, and a similar trend is observed for the ultimate strain at the loaded end.

### 3.3. Bond-Slip Relationship

The bond-slip relationship for the FFRP-to-timber interface refers to the relationship between the interfacial shear stress and the relative slip. The interfacial relative slip is defined as the displacement between the FFRP plate and the timber substrate. The bond-slip relationship of the FFRP-to-timber interface is a crucial aspect of this study and serves as the foundation for establishing a constitutive model for the interface. Therefore, analyzing the bond-slip relationship is key to investigating the interfacial bond performance of FFRP plates and timber.

There are primarily two methods to obtain the (relative) slip at the FFRP-to-timber interface: first, by direct measurement using displacement transducers (LVDTs); second, by calculation from the axial strain of the FFRP plate recorded during the test. Considering that the relative displacement between the FFRP plate and the timber substrate is very small in practice, and that direct measurement of slip using LVDTs mounted on the specimen may introduce data deviations due to various practical constraints, the second method was adopted in this study to calculate the interfacial relative slip, as shown in Equations (1) and (2) [27,30].

(1) $$\delta \left(x_{i}\right)=\delta \left(x_{i+1}\right)+\int_{x_{i+1}}^{x_{i}}\varepsilon (x)dx$$

(2) $$\delta_{i+\frac{1}{2}}=\sum_{i}^{n}\frac{\left(\varepsilon_{i}+\varepsilon_{i+1}\right)}{2}\Delta x_{i}$$

where

$\delta_{i+\frac{1}{2}}$: Slip between the i-th measurement point and the (i + 1)-th measurement point;

n: Total number of measurement points.

**Figure 12:**
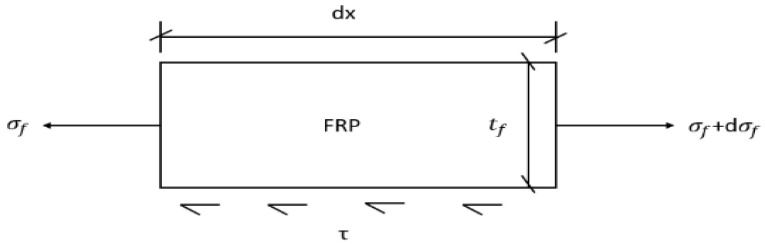
Stress analysis diagram of FRP plate.

The shear stress at the FFRP-timber interface cannot be directly measured. Typically, strain gauges are attached at unequal intervals along the FFRP plate, and the axial strain at various points on the FFRP plate is recorded during testing. The shear stress at the FFRP-timber interface is then calculated using a differential method. Although FFRP exhibits nonlinear deformation at high strain levels, the strain range used for shear stress calculation in this study (up to 0.01) remains within the approximately linear region of the FFRP stress–strain curve, as verified by preliminary tensile tests. Therefore, the linear-elastic assumption is considered acceptable for the purpose of interfacial shear stress estimation. The principle for calculating the shear stress is described below:

A differential element of length dx is considered along the FRP plate, and a detailed force analysis is illustrated in Figure 12.

Based on the force equilibrium of an infinitesimal segment (dx), the equilibrium equation is formulated as follows:

(3) $$\sigma_{f}(x)b_{f}t_{f}+\tau_{f}(x)b_{f}dx=\left[\sigma_{f}(x)+d\sigma_{f}(x)\right]b_{f}t_{f}$$

where

$\sigma_{f}$ is the axial stress in the FFRP plate; $\tau_{f}$ is the interfacial shear stress; $b_{f}$ is the width of the FFRP plate; $t_{f}$ is the thickness of the FFRP plate.

Equation (3) can be simplified to:

(4) $$\tau (x)=E_{f}t_{f}\frac{d\varepsilon (x)}{dx}$$

Although the strain measurement points are equally spaced, when the spacing is sufficiently small, the average shear stress between two adjacent points can be regarded as the shear stress at that location. That is, the interfacial shear stress between two strain points is calculated using Equation (4):

(5) $$\tau_{i+\frac{1}{2}}=E_{f}t_{f}\frac{\varepsilon_{i}-\varepsilon_{i+1}}{\Delta x_{i}}$$

where

$\tau_{i+\frac{1}{2}}$ is the shear stress at the midpoint between the i-th and the (i + 1)-th measurement points; $\varepsilon_{i}$ is the strain at the i-th measurement point; $\varepsilon_{i+1}$ is the strain at the (i + 1)-th measurement point; $E_{f}$ is the elastic modulus of the FFRP plate; $t_{f}$ is the thickness of the FFRP plate; $\Delta x_{i}$ is the spacing between the i-th and the (i + 1)-th measurement points.

For each FFRP laminate configuration, the corresponding experimentally determined elastic modulus and thickness (Table 5) were used in Equation (5) to ensure accurate estimation of interfacial shear stress.

**Figure 13:**
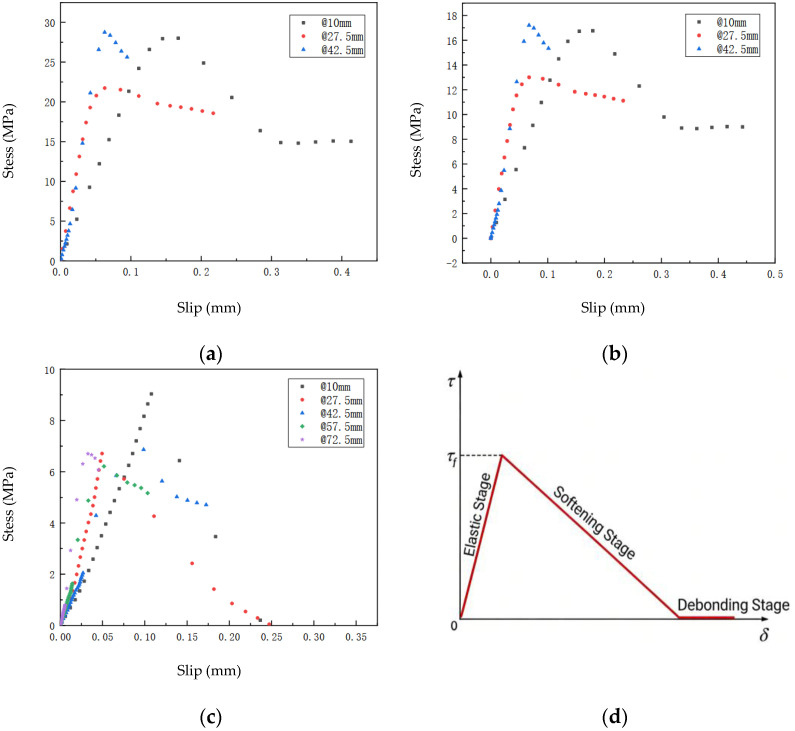
Interfacial bond-slip relationship (Data points represent mean values). (**a**) F-T-0.5. (**b**) F-T-1. (**c**) F-T-3. (**d**) Idealized Linear Adhesive T1 Bonding-Slip Relationship.

Figure 13a–c present the interfacial bond-slip relationship diagrams for FFRP plates bonded to timber using T1 adhesive, with adhesive thicknesses of 0.5 mm, 1 mm, and 3 mm, respectively. In the figures, the “@” symbol indicates the distance from the loading end. For example, “@10 mm” denotes the bond-slip scatter plot at a point 10 mm from the loading end. By comparing the interfacial bond-slip relationships of each specimen, it can be observed that the overall shapes of the curves are broadly similar. The maximum interfacial shear stress for the 0.5 mm and 1 mm thick adhesive layers is around 20 MPa, with a corresponding maximum interfacial slip of 0.4 mm. In contrast, for the 3 mm thick adhesive layer, both the maximum shear stress and maximum slip decrease, measuring 9 MPa and 0.25 mm, respectively. This indicates that the peak shear stress of the specimen decreases as the adhesive thickness increases. Specifically, as the adhesive thickness increases from 0.5 mm to 3 mm, the maximum interfacial shear stress decreases from 28.75 MPa to 9.04 MPa, representing a reduction of 68.56%. Furthermore, from Figure 13a–c, it can be observed that the bond-slip relationship for the FFRP-timber interface can be primarily divided into three stages:

Stage 1: Ascending Branch. The interfacial shear stress increases with the relative slip, and the interfacial deformation is elastic. This stage concludes when the interfacial shear stress reaches its maximum value, marking the transition to the next stage.

Stage 2: Descending (Softening) Branch. Upon reaching the peak shear stress, softening occurs. As loading continues, the shear stress gradually decreases while the slip continues to increase.

Stage 3: Frictional (Plateau) Branch. Interface debonding occurs, and the slope of the curve approaches zero. A simplified representation of this three-stage FFRP-timber interfacial bond-slip relationship is shown in Figure 13d.

### 3.4. Interfacial Stress Distribution

**Figure 14:**
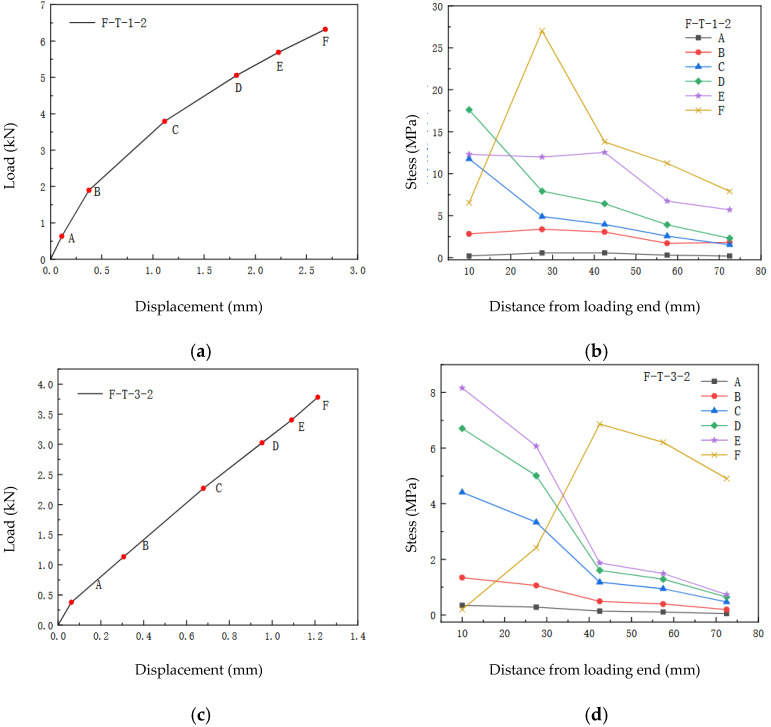
Interface stress distribution. (**a**) Load–Displacement relationship of specimen F-T-1. (**b**) Interface stress distribution of specimen F-T-1. (**c**) Load–Displacement relationship of specimen F-T-3. (**d**) Interface stress distribution of specimen F-T-3.

The average bond shear stress between two adjacent strain measurement points was calculated using the aforementioned Equation (5). The distribution of interfacial shear stress along the bonded region of the FFRP plate for the specimens is presented in Figure 14b,d. In these figures, the horizontal axis represents the distance from the loading end, while the vertical axis represents the shear stress at the midpoint between two adjacent strain points. To accurately analyze the shear stress state at each measurement point under different load levels, the variation in shear stress values at each strain point with increasing load was plotted, as shown in Figure 14a,c.

It should be noted that the values presented in the graphs are the average shear stresses between two adjacent strain points. This distribution follows the same trend as the actual interfacial shear stress distribution. Furthermore, the calculation of the average shear stress based on the strain values of the flax fiber reinforced polymer (FFRP) plate neglects any strain variation along the thickness direction of the FFRP plate.

Markers A–F: These points represent key load levels of 0.1 Pu, 0.3 Pu, 0.6 Pu, 0.8 Pu, 0.9 Pu, and 1.0 Pu selected during loading, used to analyse the mechanical behaviour of the interface at different stages.

For specimen F-T-1, the distribution of interfacial shear stress during the loading process is shown in Figure 14a,b. From Figure 14a,b, it can be observed that at the initial stage of loading (segment OA), the interfacial shear stress is relatively low across the entire bond length from the left (loaded end) to the right (free end), exhibiting a linearly decreasing trend. The shear stress near the free end, farther from the loaded end, is essentially zero. As the load continues to increase (segment AB), the interfacial shear stress also rises. When the load reaches the value at point C, it can be seen that the interfacial shear stress gradually propagates towards the free end. The shear stress curve near the loaded end becomes significantly steeper. During the increase from load C to D, the shear stress at the loaded end continues to grow, reaching its maximum value when the load attains level D. Subsequently, even as the load continues to increase, the shear stress at the loaded end begins to decrease, while the rate of increase in shear stress at the free end becomes more pronounced, as shown by curve E. When the load approaches the ultimate load (process E to F), the interfacial shear stress near the loaded end, specifically at x = 10 mm, drops to its lowest level. The peak shear stress shifts significantly from the loaded end towards the free end. The FFRP-timber interface begins to soften until final debonding failure occurs. This observed behavior aligns with findings from existing studies [27,31], confirming the reliability of the experimental results presented in this paper.

For specimen F-T-3, the interfacial stress distribution pattern, illustrated in Figure 14c,d, is fundamentally consistent with that of specimen F-T-1. Prior to reaching the ultimate load, the interfacial shear stress decreases progressively from the loaded end to the free end. When the shear stress at the loaded end reaches its maximum, the interface near this region softens, and its capacity to bear shear stress rapidly diminishes to a minimum. In contrast, the region near the free end, which has not yet softened, continues to sustain tensile forces, leading to an increase in shear stress in that area. Consequently, the stress distribution curve assumes a roughly triangular shape.

### 3.5. Interface Fracture Energy

The analysis of the FRP-wood interfacial bond-slip curve is fundamentally based on the interfacial fracture energy. Accurate calculation of the interfacial fracture energy is essential for reliably predicting the maximum load transferred to the FRP composite. Its value is equal to the area enclosed by the interfacial bond-slip curve and the *x*-axis. Based on fracture mechanics theory, Vos [32] proposed a formula for calculating the interfacial fracture energy of shear specimens:

(6) $$G_{f}=\frac{P_{u}^{2}}{2E_{f}t_{f}b_{f}^{2}}$$

where $P_{u}$ is the Ultimate load-bearing capacity of the test specimen interface (kN); $b_{f}$ is the Width of the FRP plate (mm); $E_{f}$ is the Elastic modulus of the FRP plate (GPa); $t_{f}$ is the Thickness of the FRP plate (mm).

**Figure 15:**
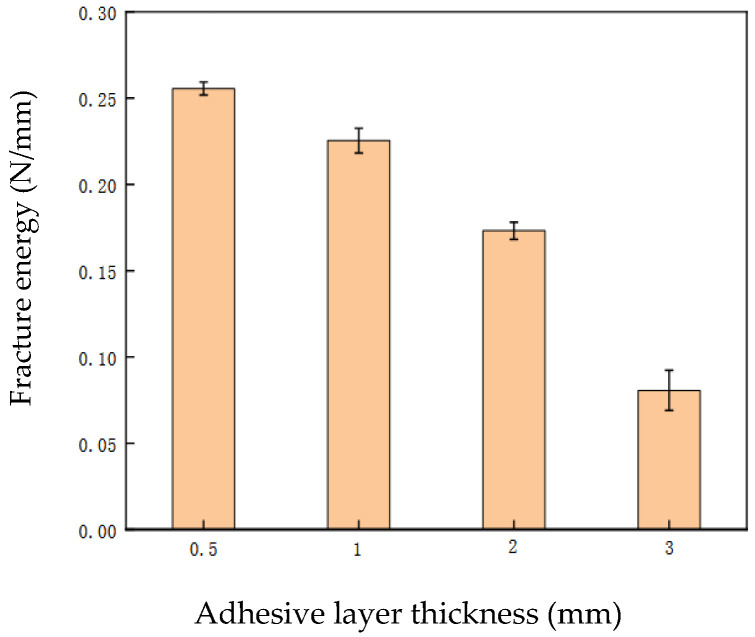
Effect of adhesive layer thickness on fracture energy of FFRP-wood interface.

Interfacial fracture energy represents the intensity of energy released during interfacial failure. A higher value of interfacial fracture energy indicates greater energy dissipation capacity of the interface, while a lower value suggests less susceptibility to damage during testing. Factors influencing interfacial fracture energy include loading rate, wood properties, type of fiber sheet, and adhesive characteristics. This study primarily investigates the effects of adhesive layer thickness, adhesive performance, and fiber sheet type on interfacial fracture energy. Through single-lap shear tests of FRP-wood interfaces, the factors influencing FRP-wood interfacial fracture energy are explored. The ultimate load-bearing capacity of the specimen interface has been listed in Table 6, and the corresponding interfacial fracture energy for each specimen was calculated using Equation (6). Figure 15 presents a comparative diagram of the interfacial fracture energy for FFRP-wood interface specimens.

As can be seen from Figure 15, under the same conditions of fiber sheet type and dimensions (length, width, and thickness), bond length, adhesive type, and loading rate, the interfacial fracture energy gradually decreases with increasing adhesive layer thickness. For example, when the adhesive layer thickness is 0.5 mm, the average interfacial fracture energy is 0.2518 N/mm, which is approximately 10.76% higher than that of the interface with an adhesive layer thickness of 1 mm, about 45.40% higher than that of the interface with a thickness of 2 mm, and 2.73 times that of the interface with a thickness of 3 mm. This indicates that a thinner adhesive layer results in greater interfacial fracture energy, a trend consistent with existing research findings on the effect of T1 adhesive thickness on the performance of CFRP-steel interfaces [33]. Figure 15 also shows that, for a given adhesive (T1), the rate of decrease in interfacial fracture energy becomes more pronounced as the adhesive layer thickness increases. The energy absorbed during interfacial failure is reflected by the interfacial fracture energy. The relationship between interfacial fracture energy and adhesive layer thickness, as illustrated in Figure 15, corroborates the variation in ultimate load-bearing capacity with adhesive layer thickness and can also be interpreted as a positive correlation between the ultimate load-bearing capacity of the interface and the fracture energy.

## 4. Influence of FFRP Plate Thickness on Interfacial Bonding Performance

### 4.1. Ultimate Load-Bearing Capacity and Failure Mode of the Interface

**Figure 16:**
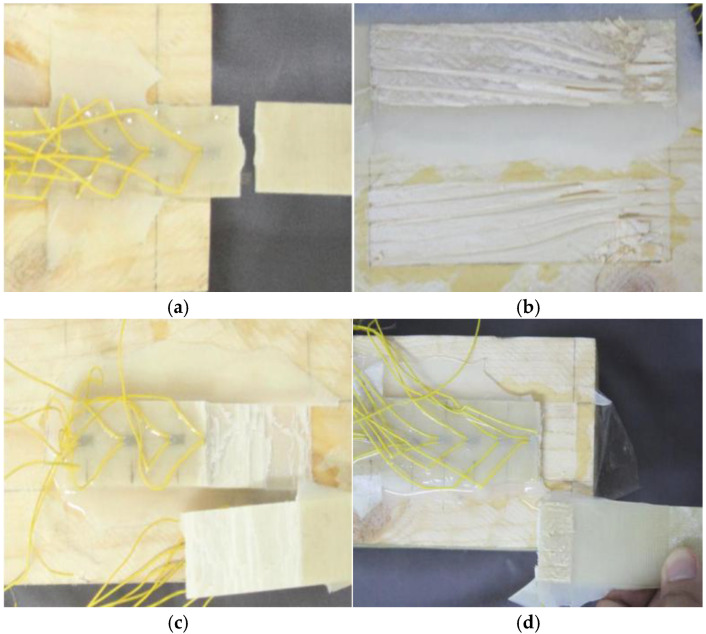
Four failure modes. (**a**) Fracture of FFRP plates. (**b**) Failure of timber. (**c**) Delamination fracture of FFRP plates. (**d**) FFRP rupture failure and timber failure.

Figure 16 illustrates four distinct failure modes: (a) FFRP plate fracture failure (hereinafter referred to as Mode R); (b) wood failure (hereinafter referred to as Mode T); (c) FFRP plate interlaminar failure (hereinafter referred to as Mode I); and (d) FFRP plate fracture combined with wood failure (R + T).

As the number of fiber layers increased, the failure mode gradually transitioned from Mode R to Modes T and I (as shown in Table 11). In Mode R, failure occurred due to insufficient tensile strength of the FFRP, resulting in fracture of the FFRP during the tension process. In Mode T, failure occurred within the wood, characterized by tearing along the grain direction; this mode, where the FFRP satisfies the interface requirements, is considered an ideal failure mode. Mode I represents a new type of failure, involving interlaminar delamination along the thickness direction of the plate [34]; in this mode, the FFRP underwent interlaminar cracking in the plastic stage, as the interlaminar strength was lower than the interface strength. For the R + T mode, failure occurred approximately 10 mm from the bonded interface, characterized by FFRP fracture accompanied by partial tearing of the wood, indicating that the FFRP strength was slightly lower than the interface strength.

It can be seen that improving the tensile properties of the FFRP helps to enhance the overall strength of the interface; however, a reduction in interlaminar strength can induce Mode I failure. Furthermore, the 30-layer FFRP-wood interface exhibited multiple failure modes, indicating a degree of instability, yet the increase in interface strength demonstrated the feasibility of reinforcing wood with 30 layers of FFRP.

The effective bond length and strain efficiency of the specimens are presented in Table 11. The effective bond length was defined as the axial length of the interface over which the interfacial strain reached or exceeded 3% of the ultimate strain. Given that the strain gauges on the interface were densely distributed, interpolation was employed to calculate the interfacial strain. The strain efficiency was defined as the ratio of the FFRP fracture strain to its ultimate tensile strain.

As the number of fiber layers increased, the effective bond length of the FFRP increased initially and then decreased, while the strain efficiency exhibited a decreasing trend followed by an increase. Specimen FFRP-A-10-T failed in Mode R with low strain efficiency, indicating that FFRP-A-10 is unsuitable for wood reinforcement. From FFRP-A-30-T to FFRP-A-50-T, the increase in ultimate load capacity of the interface was not significant, and the addition of fiber layers primarily served to stabilize the failure mode and slightly enhance the strain efficiency. Considering the trade-off between the cost and performance associated with increasing the number of FFRP fiber layers, FFRP-A-30-T represents the optimal choice.

### 4.2. Interfacial Strain Distribution

The axial strain in the FFRP plate was measured using electrical resistance strain gauges attached to its surface. The strain values extracted by the data acquisition system were arranged according to the sequence of the strain gauges, allowing for a clear observation of the distribution pattern. As the load increases, the strain also rises. To better analyze the interfacial strain distribution, the load was divided into stages. Taking the ultimate load (Pu) as a reference, stages were defined at 0.1 Pu, 0.3 Pu, 0.6 Pu, 0.8 Pu, 0.9 Pu, and 1.0 Pu. The first three load stages describe the strain distribution during the stable working phase of the interface, while the latter three describe the distribution when the interfacial properties are fully manifested.

**Figure 17:**
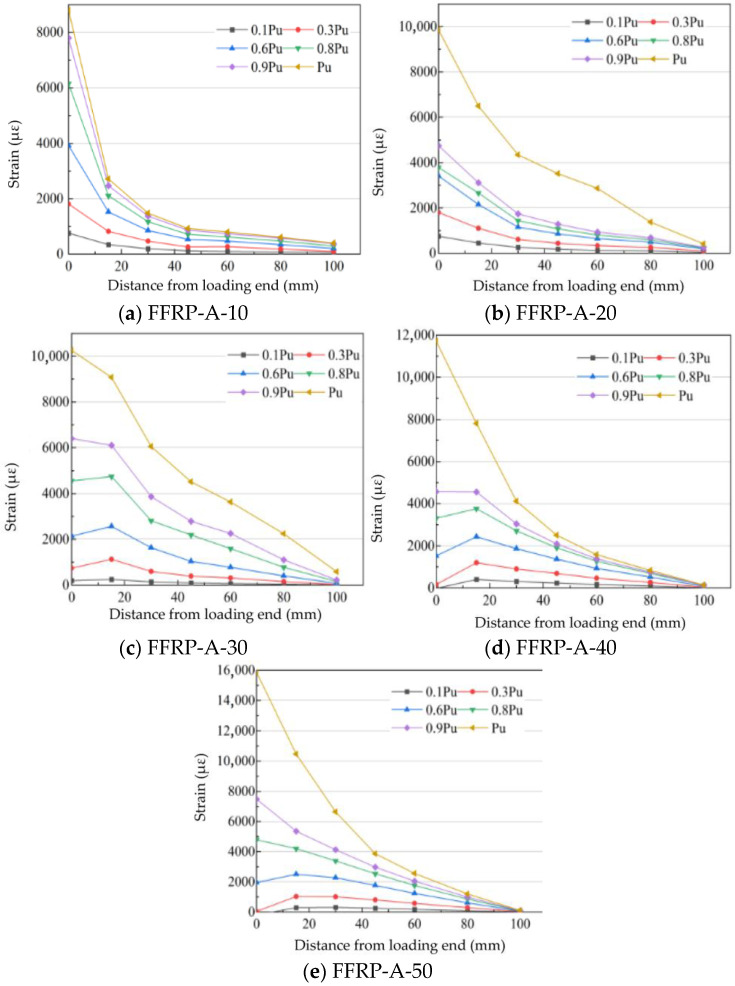
Strain distribution at the FFRP-timber structure interface.

The loading point was set as the origin (x = 0). The coordinate ‘x’ represents the distance from the loaded end; for example, x = −20 mm indicates a point 20 mm from the loaded end on the designated side. The measurement points were located along the specimen’s centerline at distances of −20 mm, 0 mm, 15 mm, 30 mm, 45 mm, 60 mm, 70 mm, and 90 mm from the loaded end. The strain distribution in the FFRP plate under these incremental load levels is shown in Figure 17, where the horizontal axis represents the distance from the loaded end and the vertical axis represents the strain (με).

For FFRP-timber structures where the FFRP strength meets the interfacial requirements (specimen FFRP-A-10-50), the observed strain distribution patterns are as follows: During the loading process from 0 to Pu, stress transfer can be divided into three stages: (1) Stress development within the FFRP plate: The fibers within the FFRP plate are subjected to tension. The interfacial strain remains very low, indicating minimal participation of the interface in load-bearing at this stage. (2) Second stage: Shear stresses develop at the interface between the natural fibers and the resin matrix within the FFRP plate. This manifests as the plate reaching its elastic limit and initiating plastic deformation. (3) Third stage: Strain decreases significantly from the free end, indicating high strain transfer efficiency at the loaded end. ultimately, debonding initiated from both ends leads to interfacial failure.

The influence of different FFRP ply numbers (layer counts) on the interfacial strain distribution is shown in Figure 17a–e. The maximum strains recorded in the single-lap shear tests for FFRP plates with 10, 20, 30, 40, and 50 fiber ply layers were 8945 µε, 9855 µε, 10,825 µε, 11,716 µε, and 15,867 µε, respectively. This increase in strain demonstrates that the interfacial ductility improves with an increasing number of fiber plies in the plate. In terms of transfer efficiency, the strain transfer efficiency initially increases and then decreases as the number of fiber plies in the FFRP plate increases. The maximum strain transfer efficiency is achieved when the plate contains 30 fiber ply layers.

### 4.3. Bond-Slip Relationship

**Figure 18:**
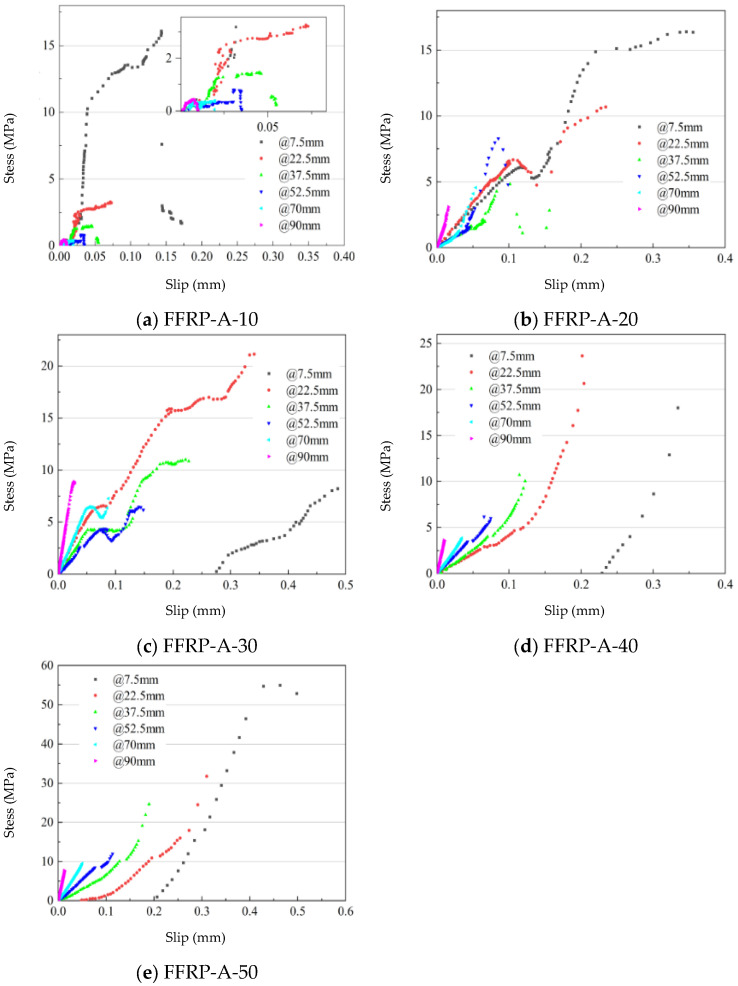
Bond-slip relationship at the FFRP-timber structure interface (Data points represent mean values).

As shown in Figure 18, the ultimate slip values for specimens FFRP-A-10, FFRP-A-20, FFRP-A-30, FFRP-A-40, and FFRP-A-50 are 0.172 mm, 0.249 mm, 0.486 mm, 0.334 mm, and 0.498 mm, respectively. The interfacial ultimate slip generally increases with the number of fiber plies in the FFRP plate.

As the number of fiber plies in the FFRP increases, the centroid of the interfacial bond-slip relationship shifts from 7.5 mm to 22.5 mm, indicating a gradual enhancement in interfacial stress transfer. Comparing FFRP-A-10 and FFRP-A-20, within the slip range of 0.1–0.15 mm, the bond-slip curve for FFRP-A-20 at 7.5 mm from the loaded end exhibits a plateau stage, representing the softening phase of the interface. In contrast, at the same location for FFRP-A-10, the interface shows increased plasticity, reflecting more of the nonlinear large-deformation characteristics of the FFRP-A plate. As the number of fiber plies increases from 10 to 20, the interfacial behavior incorporates a more pronounced softening stage, and both stress transfer and ductility tend to improve (ultimate slip: FFRP-A-10 = 0.172 mm, FFRP-A-20 = 0.249 mm).

Comparing FFRP-A-20 and FFRP-A-30, at 37.5 mm from the loaded end and after a slip of 0.1 mm, the stress in FFRP-A-20 begins to decline, whereas FFRP-A-30 shows a stress increase followed by hardening. These characteristics are also observed at distances of 52.5 mm and 70 mm from the loaded end. The overall integrity between the FFRP-A plate and the reinforced interface improves, leading to enhanced interfacial performance. Additionally, the bond-slip curve at 7.5 mm from the loaded end for FFRP-A-30 is only observed when the slip reaches 0.27 mm. This may be attributed to increased fiber interactions between layers and interlayer displacements in plates with higher ply counts, resulting in missing slip observations.

Comparing FFRP-A-30 and FFRP-A-40, the missing slip observation at 7.5 mm persists for FFRP-A-40, indicating that once the ply count reaches 30, the FFRP-A plate can generate interfacial slip, leading to a slower slip transfer process. Unlike FFRP-A-30, the bond-slip behavior of FFRP-A-40 is predominantly linear. The softening stage observed in FFRP-A-30 shortens to a mere point in FFRP-A-40—still present but significantly reduced. Consequently, the ductility of FFRP-A-40 decreases compared to FFRP-A-30 (ultimate slip: FFRP-A-30 = 0.486 mm, FFRP-A-40 = 0.334 mm).

Comparing FFRP-A-40 and FFRP-A-50, the maximum stress increases from 23.64 MPa to 54.95 MPa. The centroid of the bond-slip relationship shifts from 22.5 mm in FFRP-A-40 to 7.5 mm in FFRP-A-50, and the ductility increases to a level similar to that of FFRP-A-30. For FFRP-A plates with more than 20 plies, only the ascending branch of the bond-slip curve is clearly observable, while the descending branch is largely absent. This is attributed to internal interactions within the FFRP-A plate due to the higher ply count, which restricts the strain transferred to the interface.

As the number of fiber plies in FFRP-A increases from 10 to 30, the centroid of the interfacial bond-slip relationship shifts from 7.5 mm to 22.5 mm from the loaded end, indicating optimal interfacial performance. For ply counts of 40 and 50, the softening stage in the bond-slip curve diminishes, the linear stage becomes more prominent, and the centroid shifts back to 7.5 mm from the loaded end. In terms of stress transfer efficiency, ultimate slip, and maximum stress, both FFRP-A-30 and FFRP-A-50 exhibit favorable interfacial performance, though their bond-slip relationships differ significantly.

### 4.4. Interfacial Stress Distribution

**Figure 19:**
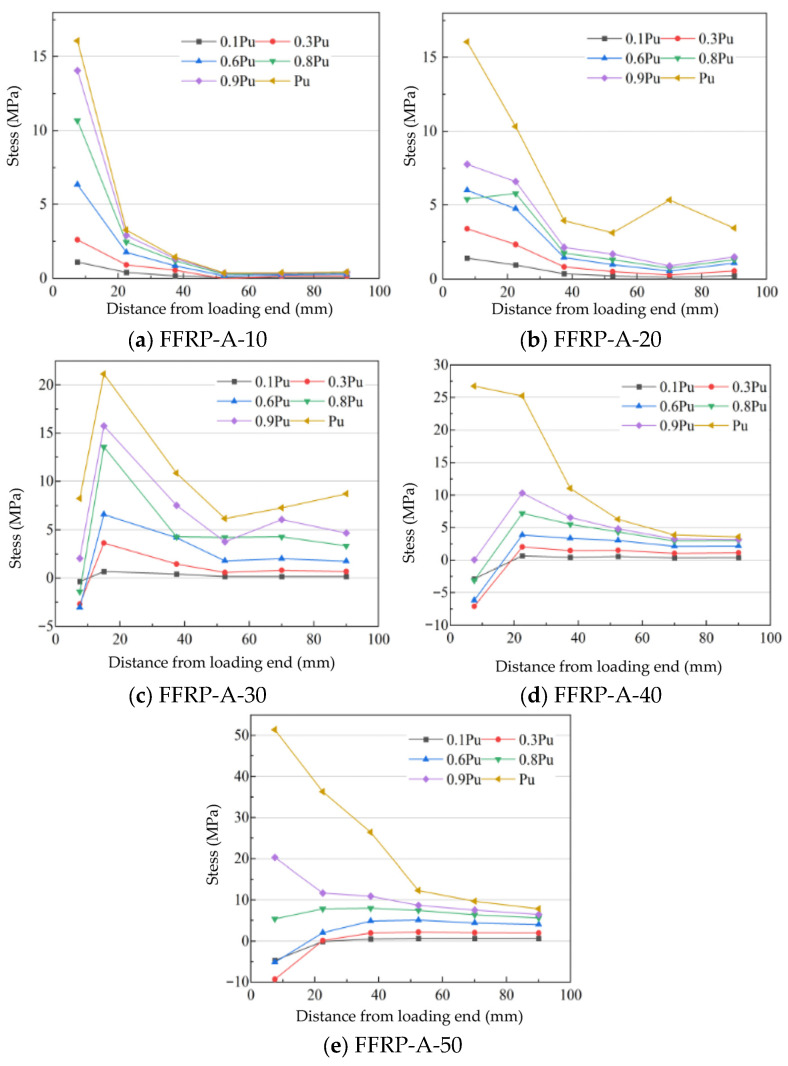
Stress distribution at the FFRP-timber structure interface.

The interfacial shear stress in the FFRP-timber structure can be calculated using Equation (5). Figure 19 presents the distribution curves of interfacial shear stress along the axial direction from the loaded end to the free end under different load levels. Based on the observed stress distribution patterns, this study categorizes the interfacial shear stress distributions into four types.

(1) Continuously decreasing distribution, this pattern is characterized by an increase in interfacial shear stress near the loaded end as the load level rises. However, the magnitude of this increase diminishes with greater distance from the loaded end. Near the free end, variations in load level produce almost no difference in stress. This distribution primarily occurs when the plate strength is low, leading to inefficient interfacial stress transfer. The FFRP plate primarily bears tensile stress, which further reduces the efficiency of stress transfer across the interface.
(2) Smooth transfer distribution, at low load levels, the stress decreases smoothly and gradually from the free end to the loaded end, with a very small reduction gradient. Under high load levels, however, the stress is highest near the free end and decreases in stages with increasing distance from the loaded end, showing a significant reduction gradient. At a distance of 52.5 mm from the loaded end, the reduction in interfacial stress diminishes, and the transfer becomes smooth again. This distribution pattern is observed in cases such as FFRP-A-20, FFRP-A-40, and FFRP-A-50.
(3) Bimodal stress distribution, this interfacial shear stress distribution features both a high peak and a low valley. At low load levels, the distribution conforms to the smooth transfer type. As the load level increases, the interfacial shear stress gradually rises near both the free end and the loaded end, forming two stress peaks with a boundary around 52.5 mm from the loaded end. During the load increase, the changes are gradual, exhibiting better ductility and higher interfacial strength. This distribution pattern is applicable to cases like FFRP-A-30.
(4) Unimodal stress distribution, this pattern indicates high interfacial transfer efficiency, and the overall distribution still follows a smooth transfer trend. However, as the load increases, the interfacial transfer efficiency gradually improves, forming a single peak distribution near the free end. This signifies that stress is fully transferred to the interface at the free end. The fact that the stress at the loaded end is lower than at the free end suggests that interface failure initiates at the free end.

The interfacial stress in the FFRP plate exhibits a continuously decreasing distribution pattern. For FFRP-A-10, the trend of interfacial stress distribution remains consistent across various load levels; however, the stress transfer efficiency decreases as the load increases. Variations in the number of fiber plies in the FFRP plate lead to differences in interfacial performance. Taking FFRP-A-10 and FFRP-A-20 as examples, as the number of fiber plies in the FFRP material increases, the interfacial stress distribution transitions from the original continuously decreasing type to a smooth transfer type. Specifically, in the region beyond 40 mm from the loaded end, the stress transfer efficiency improves, while the maximum interfacial stress value remains unchanged (the shear stress for both FFRP-A-10 and FFRP-A-20 is 16.06 MPa). This indicates that increasing the number of fiber plies in the FFRP-A plate from 10 to 20 does not alter the maximum interfacial stress but does influence the state of stress distribution. When the number of fiber plies in FFRP-A increases to 30, the interfacial stress distribution presents a bimodal pattern, with stress peaks observed near both the loaded end and the free end. At the ultimate load (Pu), the maximum interfacial shear stress near the loaded end is 22.31 MPa, and a secondary peak of 8.72 MPa occurs at 90 mm from the loaded end. The shear stress acting at both ends contributes to interfacial failure. This demonstrates that with 30 plies in FFRP-A, the interfacial stress distribution becomes bimodal, exhibiting high transfer efficiency, which reflects favorable interfacial performance. When the number of fiber plies in FFRP-A increases to 50, the stress distribution reverts to a smooth transfer type, and the interfacial stress transfer efficiency decreases compared to that of FFRP-A-30. Unlike FFRP-A-20, the maximum stress increases with the number of fiber plies in FFRP-A (rising to 23.64 MPa for FFRP-A-40 and 51.39 MPa for FFRP-A-50), while the transfer efficiency declines.

### 4.5. Interface Fracture Energy

Based on Equation (6), the interfacial fracture energy was calculated, and the results are as follows: FFRP-A-10 (0.37 N/mm); FFRP-A-20 (0.74 N/mm); FFRP-A-30 (1.53 N/mm); FFRP-A-40 (1.21 N/mm); FFRP-A-50 (1.62 N/mm).

As the number of fiber layers in the FFRP-A plate increased, the interfacial fracture energy of the FFRP-A-20 and FFRP-A-30 wood interfaces reached 1.97 times (0.74/0.37) and 4.11 times (1.53/0.37) that of the FFRP-A-10 interface, respectively. When the number of FFRP-A fiber layers reached 30, the fracture energy of the FFRP-wood interface tended to stabilize. The interfacial fracture energy of the FFRP-A-40 plate-wood interface decreased, though only slightly, while that of the FFRP-A-50 plate-wood interface was similar to that of FFRP-A-30.

## 5. Conclusions

This study investigated the interfacial bond performance between FFRP and timber through two distinct single-lap shear test series, examining the effects of different adhesive thicknesses and varying numbers of FFRP plies. The main findings are summarized as follows:

(1) The test results indicate that the ultimate interfacial load-carrying capacity decreases as the adhesive thickness increases. A greater adhesive thickness which reduces relative sliding between bonded surfaces and diminishes the deformation energy absorbed by the interface, thereby weakening its load-bearing capacity. When the adhesive layer thickness is within the range of 0.5 mm to 1.5 mm, the reduction in interfacial capacity is not significant. This range can therefore be defined as the optimal adhesive thickness.
(2) Analysis of the axial strain distribution in the FFRP plate reveals that a greater adhesive thickness results in a smaller ultimate strain value. During the initial loading stage, the peak shear stress occurs primarily at the loaded end, and the interfacial shear stress decreases gradually from the loaded end towards the free end. As the applied load increases, the peak interfacial shear stress shifts from the loaded end towards the free end.
(3) Material property tests on FFRP with different numbers of fiber plies show that as the number of plies increases, the tensile modulus, tensile strength, and interlaminar strength of the FFRP decrease. The elongation at break first decreases and then increases, with higher values observed for FFRP with 10 and 30 plies.
(4) The FFRP-timber interface with FFRP-A-10 exhibits relatively inefficient interfacial performance. As the number of fiber plies increases, the interfacial slip gradually increases, and the interface demonstrates enhanced ductility and improved shear resistance. However, significant differences are observed in the interfacial bond-slip relationships. FFRP-A-50 exhibits the highest interfacial strength and stiffness, followed by FFRP-A-30. Although FFRP-A-50 exhibits higher peak stress, FFRP-A-30 demonstrates superior strain transfer efficiency and ductility, making it more suitable for applications requiring balanced interfacial performance. Among the configurations tested, FFRP-A-30 is deemed more suitable for timber reinforcement, as it provides the interface with greater load capacity and tensile strength.

## Figures and Tables

**Table 1:** Basic material parameters of timber.

Timber Species	Moisture Content (%)	Density (kg/m^3^)	Tensile Strength (MPa)	Flexural Strength (MPa)	Tensile Modulus (GPa)
Russian pine timber	13.6	462	34.3	65.2	8.9

**Table 2:** Mechanical properties of FFRP.

Sample Types		Tensile Strength (MPa)	Modulus of Elasticity (GPa)	Nominal Thickness (mm)	Elongation at Break (%)
FFRP	Longitude Latitude	47.03 (±17.2) 115.21 (±41.44)	21.41 (±0.62) 48.50 (±1.96)	2.05 2.07	3.26 (±1.06) 1.13 (±0.16)

**Table 3:** T1 epoxy structural adhesive parameters.

Adhesive	Tensile Strength (MPa)	Tensile Modulus (MPa)	Elongation at Break (%)	Viscosity Value (25 °C) (cps)		Color, Shape		Curing Time (h)
				A	B	A	B	
T1	50	3.13	1.6	1000	2000	Transparent or egg-yolk colored, viscous colloid	Clear, irritating liquid	3~6

**Table 4:** Parameters of specimens (mean).

Specimen Number	Adhesive Layer Thickness (mm)	Types of Adhesives	Sheet Material Types	t (mm)	b (mm)	Number of Replicates
F-T-0.5	0.5	T1	FFRP	2.05	30	3
F-T-1	1	T1	FFRP	2.05	30	3
F-T-2	2	T1	FFRP	2.05	30	3
F-T-3	3	T1	FFRP	2.05	30	3

Note: t denotes the plate thickness; b denotes the plate width. The specimen designation follows the nomenclature I-II-III, where I indicates the plate type, II indicates the adhesive type, and III indicates the adhesive layer thickness.

**Table 5:** Parameters of specimens (mean).

Specimen Number	Tensile Strength (MPa)	$E_{p}$ (GPa)	$t_{n}$ (mm)	$t_{p}$ (mm)	Elongation at Break (%)	Number of Replicates
FFRP-A-10	173.17	19.79	2.00	0.88	1.56	3
FFRP-A-20	167.20	19.31	3.72	1.75	1.32	3
FFRP-A-30	154.01	18.48	5.58	2.63	1.53	3
FFRP-A-40	111.64	15.18	6.86	3.50	1.38	3
FFRP-A-50	120.35	16.30	8.89	4.38	1.28	3

Note: The specimen designation follows the format I-II-III, where I denotes the type of plate, II denotes the manufacturing process (with A representing the autoclave process), and III denotes the number of layers in the plate.

**Table 6:** Test result (mean).

Specimen Number	Adhesive Layer Thickness (mm)	Types of Adhesives	$P_{u,a}$(kN)	Interface Destruction Mode	Number of Replicates
F-T-0.5	0.5	T1	6.73	a	3
F-T-1	1	T1	6.32	d/a/e	3
F-T-2	2	T1	5.54	a + e	3
F-T-3	3	T1	3.78	e/c	3

Note: The thickness of the adhesive layer refers to the ideal bonding thickness of the adhesive; $P_{u,a}$ represents the average ultimate load-bearing capacity of the specimen interface. a denotes fracture failure of the FFRP plate, c refers to adhesion failure at the interface between the FFRP plate and the adhesive (i.e., the upper surface of the adhesive layer), d indicates adhesion failure at the interface between the adhesive (i.e., the lower surface of the adhesive layer) and the wood, and e represents peeling failure of the wood.

**Table 7:** Axial strain of FFRP plate under each load level of specimen F-T-0.5 (mean).

Distance from Loading End (mm)	Axial Strain of FFRP Plate (με)					
	0.1 Pu	0.3 Pu	0.6 Pu	0.8 Pu	0.9 Pu	1 Pu
0	586.26	2113.15	5467.41	9981.81	10,744.63	12,699.84
20	555.61	1987.48	4437.88	6427.01	7182.23	7658.82
35	210.05	808.59	1840.14	2606.21	3005.82	3352.46
50	111.31	440.82	978.30	1338.14	1516.31	1716.90
65	84.18	318.40	692.65	936.38	1056.68	1190.92
80	68.17	257.24	545.19	728.81	819.28	918.24
100	59.76	211.85	431.57	569.47	634.74	703.26

**Table 8:** Axial strain of FFRP plate under each load level of specimen F-T-1 (mean).

Distance from Loading End (mm)	Axial Strain of FFRP Plate (με)					
	0.1 Pu	0.3 Pu	0.6 Pu	0.8 Pu	0.9 Pu	1 Pu
0	334.87	2350.17	6086.33	8894.55	9151.94	11,405.77
20	294.96	1782.18	3719.18	5348.87	6676.04	10,091.70
35	211.13	1273.00	2981.00	4154.07	4866.96	6013.47
50	126.50	812.88	1780.85	2430.73	2975.26	3932.98
65	81.05	555.39	1392.69	1839.11	1957.52	2236.15
80	−53.22	131.57	254.99	886.73	1096.27	1044.26

**Table 9:** Axial strain of FFRP plate under each load level of specimen F-T-2 (mean).

Distance from Loading End (mm)	Axial Strain of FFRP Plate (με)					
	0.1 Pu	0.3 Pu	0.6 Pu	0.8 Pu	0.9 Pu	1 Pu
0	104.89	1679.61	4052.56	5677.10	6748.43	8463.22
20	61.64	1571.73	2665.39	3397.26	3795.03	5502.45
35	60.83	861.43	1845.98	2335.20	2614.21	3157.37
50	31.16	650.71	1188.28	1308.41	1429.40	1683.03
65	12.75	297.27	976.58	1011.38	1213.34	1551.62
80	7.69	183.67	694.75	710.50	738.86	987.30
100	2.03	61.35	56.24	28.60	131.03	403.96

**Table 10:** Axial strain of FFRP plate under each load level of specimen F-T-3 (mean).

Distance from Loading End (mm)	Axial Strain of FFRP Plate (με)					
	0.1 Pu	0.3 Pu	0.6 Pu	0.8 Pu	0.9 Pu	1 Pu
0	182.94	683.99	1974.33	2923.94	3481.56	4051.98
20	111.39	413.14	1086.86	1574.67	1839.63	4011.12
35	68.44	252.81	583.57	819.16	924.25	3645.52
50	46.94	178.11	405.27	576.56	641.01	2610.01
65	29.92	118.35	262.51	382.05	415.22	1672.97
80	21.86	88.44	190.84	283.75	304.34	932.86

**Table 11:** Single-shear test results.

Test Specimen	Resilience	Effective Bonding Length (mm)	Ultimate Load Capacity (kN)	Destruction Mode	Number of Replicates
FFRP-A-10-T	0.84	87.74	4.20	R	3
FFRP-A-20-T	0.74	92.45	5.71	R/T	3
FFRP-A-30-T	0.70	100.25	10.34	R/T/I	3
FFRP-A-40-T	0.81	97.34	8.73	T/R	3
FFRP-A-50-T	1.06	95.58	13.68	T/I	3

## Data Availability

The original contributions presented in the study are included in the article. Further inquiries can be directed to the corresponding author.
